# Auditory Hallucinations as a Rare Presentation of Occipital Infarcts

**DOI:** 10.1155/2018/1243605

**Published:** 2018-06-27

**Authors:** Firas Ido, Reina Badran, Brandon Dmytruk, Zain Kulairi

**Affiliations:** Wayne State University School of Medicine, 1101 W. University Drive, 2 South, Rochester, MI 48307, USA

## Abstract

A stroke is a clinical syndrome characterized by a focal neurologic deficit that can be attributed to a vascular territory within the brain. The presenting features of an acute stroke depends on the area of the brain affected. Although unusual, the presenting feature may include psychosis with auditory and/or visual hallucinations. A 56-year-old female was admitted to the psychiatric unit after threatening her husband with a knife. She reported experiencing altered sensorium for one week with suicidal and homicidal command hallucinations. Given the acute onset, brain images were obtained to rule out an organic etiology. A brain MRI revealed an acute right occipital lobe infarct with hemorrhagic transformation. The patient's symptoms were self-limited, resolving without antipsychotic medications. Psychosis with auditory hallucinations is not commonly reported following stroke. Since histologic and functional alterations in the occipital lobe appear to play a significant role in psychosis of schizophrenics, it is likely that ischemia in the same area may cause similar changes. Familiarity with this rare presentation is important, as it prevents a delay in diagnosis, which may negatively impact the outcome.

## 1. Background

Stroke is the fourth leading cause of mortality in the US affecting both men and women [1]. It is a clinical syndrome characterized by a focal neurologic deficit that can be attributed to a vascular territory within the brain. Stroke is organized primarily as ischemic or hemorrhagic, occurring in 85% and 15% of all cases, respectively, which can be distinguished with brain imaging [2]. The pathophysiology of ischemic stroke may involve a cardioembolic phenomenon, large artery emboli, large/small artery atherosclerosis with thrombus formation, and small artery arteriosclerosis in the setting of hypertension leading to lacunar infarcts. In contrast, hemorrhagic infarcts occur more commonly in the setting of a ruptured intracerebral aneurysm or trauma [3].

The presenting features of an acute stroke depend on the area of the brain affected by the vascular insult. Based on arterial anatomy, anterior and middle cerebral involvement commonly present with hemiparesis, hemiplegia, facial droop, dysarthria, aphasia, and gaze preference. Posterior circulatory infarcts affect the cerebellum and occipital lobe which can manifest as vertigo, ataxia, and visual disturbances [3].

Although poststroke mood and emotional disturbances such as depression, anxiety, and anger are common, psychiatric manifestations as a presenting feature of stroke are rare and limited in number of reported cases [4]. When present, psychiatric symptoms may differ depending on the area of the brain involved. Symptoms of hallucinations and delusions are uncommon but have been reported in strokes involving the caudate and thalamus [5]. Even more discrete changes in behavior from baseline such as increased sleep and not attending work were documented in a basal ganglia stroke [6]. The frontal lobe, being the primary focus for executive function, has been linked to personality changes such as emotional instability, impulsivity, and apathy [7]. In addition, the presence of visual and auditory hallucinations were noted to be a presenting feature of a right frontal stroke [8]. In a retrospective analysis, 7% of patients that presented with altered mental status were found to have an acute stroke in the frontoparietal, occipital, frontal, and right pontine areas of the brain [9]. Right temporal lobe strokes have been associated with the 5 behavioral phenomena (hypergraphia, atypical sexuality, intensified mental life, circumstantiality, and hyperreligiosity) which are the components of Geschwind syndrome [10]. Finally, strokes involving the temporooccipital regions have been documented to present with excessive talkativeness and repetitiveness, termed logorrhea [11].

Although unusual, the presenting feature of a stroke may rarely include psychosis with auditory and/or visual hallucinations in the absence of somatic alterations [12]. In the rare circumstance that the stroke does present with psychosis as the predominating symptom, this may result in a delay in diagnosis. Given the time sensitivity of imaging and early medical therapy in stroke, this delay in diagnosis may negatively impact the outcome.

## 2. Case Report

A 56 year-old female was transported to the emergency department by EMS after physically threatening her husband with a knife. According to the patient, she experienced altered sensorium for one week prior to presentation, primarily resulting in suicidal and homicidal command hallucinations instructing her to overdose on NSAIDs and kill her husband. She denied any headaches, vertigo, fevers, head trauma, urinary symptoms, or use of illicit substances. There was no history of psychosis, schizophrenia, mania, or depression and a review of her medication list for any potential hallucinogenic agents did not indicate a pharmacologic etiology. Her past medical history was comprised of two prior strokes, the most recent being two years ago that involved the right frontal lobe. MRI of the brain at the time also showed evidence of an old right parietal lobe infarct. An echocardiogram during that hospitalization revealed a severely decreased left ventricular function and the patient was initiated on warfarin for anticoagulation. Since the two prior cerebral infarcts, the patient and her husband denied noting any behavioral changes, cognitive impairment, or any focal neurologic deficits. On presentation, she appeared disheveled and exhibited a flat affect with minimal verbalization. Physical examination revealed only a left hemianopia without hemiplegia. All cranial nerves were evaluated as well as gait, cerebellar function, and proprioception, which were all found to be normal. The patient was alert and oriented with intact mentation.

**Figure 1:**
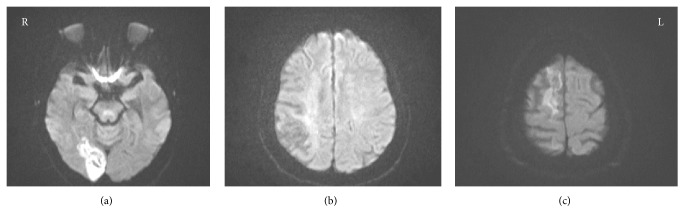
(a) Diffuse weighted/TRACE-acute right occipital infarct with hemorrhagic transformation. (b) Diffuse weighted/TRACE-encephalomalacia of the right posterior parietal lobe. (c) Diffuse weighted/TRACE-encephalomalacia of the superior frontal lobe.

She was initially admitted to the psychiatric unit for further evaluation where the patient participated in daily activities and reported no symptoms. Given the acute onset of her symptoms, laboratory studies and brain images were obtained in order to rule out an organic etiology. A complete blood count was normal and a basic metabolic panel revealed normal electrolytes and renal function. Additional labs included liver function tests, lipid panel, cardiolipin antibody, and TSH, which were all normal and RPR was nonreactive. A urine sample was negative for urinary tract infection and 8-panel urine drug screen was negative. An MRI of the brain was obtained, which revealed old ischemic infarcts within the right parietal and frontal lobes along with a new acute right occipital lobe infarct with hemorrhagic transformation (Figure 1). The patient was subsequently transferred to the medical unit for further workup and management.

The patient was placed on telemetry, which showed normal sinus rhythm. Given the involvement of multiple brain territories and circulations, a cardioembolic source of stroke was highly suspected. A cardiologist evaluated the patient and performed a transesophageal echocardiogram that revealed a low ejection fraction of less than 20%. In addition, a bubble study was performed that was positive for a patent foramen ovale. Given her significantly low ejection fraction, the patient underwent placement of an automated implantable cardioverter-defibrillator. The patient's presenting symptom of psychosis, primarily in the form of auditory hallucinations, was self-limited and resolved on day two of hospitalization without requiring the use of antipsychotic medications.

## 3. Discussion

Psychosis with auditory hallucinations is not a commonly reported behavioral alteration following stroke [13]. These symptoms are more typical features of drug intoxication and psychiatric illnesses including brief psychotic episode, schizophreniform, and schizophrenia. Symptoms of psychosis can be categorized into positive symptoms (hallucinations, delusions, and disorganized speech) and negative symptoms (flat affect and avolition) [14]. Auditory hallucinations, the presence of auditory sensation in the absence of external stimuli, have been described in less than 1% of patients found to have subcortical, brainstem, and temporal infarcts [15]. These hallucinations are described as a dissociative phenomenon of perceiving peers/relatives conversing or hearing one's own voice [16]. Command hallucinations following a stroke are atypical and therefore may be misdiagnosed as a primary psychiatric disorder.

The auditory center, which is primarily located in the temporal lobes, has been linked to psychosis in schizophrenia [17]. Auditory hallucinations in cerebrovascular disease have been observed with infarction involving the bitemporal cerebral cortex [18]. They have also been described in strokes involving the anterior/inferior cerebellar arteries and posterior circulation [18]. Therefore, it is plausible that ischemia to neurons involved in excitatory/inhibitory function may alter the sensorium leading to abnormal behaviors. The areas of the brain most commonly associated with schizophrenia include the frontal lobes, temporal lobes, limbic system, and brain stem. The occipital lobe, which is the primary visual cortex, has not been traditionally associated with auditory function. However, recent evidence regarding the role of the occipital brain in schizophrenia has showed altered volume and histologic changes including changes in density involving gray and white matter within the occipital lobes [19]. Since histologic and functional alterations in the occipital lobe appear to play a significant role in psychosis of schizophrenics, then it is likely that the ischemia that occurs following a cerebral infarct in the same area may cause similar changes.

Our patient did have a history of multiple ischemic infarcts, but did not exhibit any prior behavioral changes. Following her new occipital stroke, her only presenting feature was auditory command hallucinations. These hallucinations were self-limited and resolved the following day without any intervention. In summary, cerebral infarcts presenting with hallucinations along with other symptoms of psychosis are rare and can therefore lead to a delay in diagnosis that can have catastrophic outcomes. Familiarity with behavioral changes as a presenting sign of stroke is very important, as it allows for prompt diagnosis and early treatment. The additional signs of stroke that clinicians should be familiar with include auditory and visual hallucinations. Not only does this allow for time sensitive management to occur, but it also avoids unnecessary psychiatric treatment.
